# PANoptosis-Related Optimal Model (PROM): A Novel Prognostic Tool Unveiling Immune Dynamics in Lung Adenocarcinoma

**DOI:** 10.1155/ijog/5595391

**Published:** 2025-02-18

**Authors:** Jianming Peng, Leijie Tong, Rui Liang, Huisen Yan, Xiuling Jiang, Youai Dai

**Affiliations:** ^1^School of Medicine, Yangzhou Polytechnic College, Yangzhou, China; ^2^Department of Immunology, China Medical University, Shenyang, China; ^3^School of Basic Medical Science, Suzhou Vocational Health College, Suzhou, China; ^4^Laboratory of Organ Transplantation Research Institute, Wuxi People's Hospital Affiliated to Nanjing Medical University, Wuxi, China

**Keywords:** LUAD, PANoptosis, prognostic signature, single-cell genomics, tumor immune microenvironment

## Abstract

**Background:** PANoptosis, a recently characterized inflammatory programmed cell death modality orchestrated by the PANoptosome complex, integrates molecular mechanisms of pyroptosis, apoptosis, and necroptosis. Although this pathway potentially mediates tumor progression, its role in lung adenocarcinoma (LUAD) remains largely unexplored.

**Methods:** Through comprehensive single-cell transcriptomic profiling, we systematically identified critical PANoptosis-associated gene signatures. Prognostic molecular determinants were subsequently delineated via univariate Cox proportional hazards regression analysis. We constructed a PANoptosis-related optimal model (PROM) through the integration of 10 machine learning algorithms. The model was initially developed using The Cancer Genome Atlas (TCGA)-LUAD cohort and subsequently validated across six independent LUAD cohorts. Model performance was evaluated using mean concordance index. Furthermore, we conducted extensive multiomics analyses to delineate differential pathway activation patterns and immune cell infiltration profiles between PROM-stratified risk subgroups.

**Results:** Cellular populations exhibiting elevated PANoptosis signatures demonstrated enhanced intercellular signaling networks. PROM demonstrated superior prognostic capability across multiple validation cohorts. Receiver operating characteristic curve analyses revealed area under the curve values exceeding 0.7 across all seven cohorts, with several achieving values above 0.8, indicating robust discriminative performance. The model score exhibited significant correlation with immunological parameters. Notably, high PROM scores were associated with attenuated immune responses, suggesting an immunosuppressive tumor microenvironment. Multiomics investigations revealed significant alterations in critical oncogenic pathways and immune landscape between PROM-stratified subgroups.

**Conclusion:** This investigation establishes PROM as a clinically applicable prognostic tool for LUAD risk stratification. Beyond its predictive utility, PROM elucidates PANoptosis-associated immunological and biological mechanisms underlying LUAD progression. These findings provide novel mechanistic insights into LUAD pathogenesis and may inform the development of targeted therapeutic interventions and personalized treatment strategies to optimize patient outcomes.

## 1. Introduction

Lung cancer remains one of the most prevalent and deadly malignancies worldwide, with over 2.2 million new cases and approximately 1.8 million deaths reported annually. Non-small cell lung cancer (NSCLC) constitutes over 85% of all lung cancer cases, with lung adenocarcinoma (LUAD) representing the predominant histological subtype, accounting for nearly 40% of NSCLC cases [1, 2]. LUAD is characterized by its distinct molecular profiles, diverse genomic alterations, and complex tumor microenvironment, which contribute to its heterogeneous clinical manifestations and treatment responses. Current therapeutic approaches for LUAD encompass surgery, targeted therapy, radiotherapy, immunotherapy, and chemotherapy. Despite substantial advancements in diagnosis and treatment, including the development of targeted therapies against driver mutations such as EGFR, ALK, and ROS1, the prognosis for LUAD patients remains grim, with a 5-year survival rate still below 20% [3]. The pronounced heterogeneity, high recurrence rates, and metastatic potential of LUAD present formidable challenges that diminish the effectiveness of existing therapies [4]. Therefore, there is an urgent need to explore novel pathways and molecular mechanisms, such as PANoptosis, for identifying biomarkers associated with prognosis and therapeutic response in LUAD patients. Uncovering such biomarkers is crucial for advancing precision medicine in lung cancer, ultimately aiming to improve survival outcomes for those affected by LUAD.

PANoptosis is a unique inflammatory mechanism of programmed cell death (PCD) that operates through the formation of the PANoptosome complex, integrating features of pyroptosis, apoptosis, and necroptosis [5]. This process is essential in enabling cells to defend against various stressors and combat pathogenic threats. PANoptosis is closely linked to a spectrum of diseases, including infections, sterile inflammation, and cancer [6]. The PANoptosome serves as the central regulatory complex of PANoptosis, composed of key components such as the NLRP3 inflammasome (which initiates pyroptosis), RIPK3 (which is crucial for necroptosis), and CASP8 (which triggers apoptosis) [7]. Upon encountering pathogenic infections, inflammatory signals like TNF-*α*, or various stressors, the PANoptosome assembles, leading to the activation of specific proteases and the initiation of the cell death process. The activation of the PANoptosome triggers a coordinated response involving pyroptosis, apoptosis, and necroptosis, characterized by gasdermin D (GSDMD) cleavage, which forms pores in the cell membrane, CASP8 activation that results in nuclear fragmentation and DNA degradation, and the involvement of RIPK3 and MLKL, which destabilize membrane integrity, ultimately leading to cell lysis [8]. Besides inducing cell death, PANoptosis amplifies local inflammatory responses by releasing damage-associated molecular patterns (DAMPs) and various inflammatory cytokines, which subsequently stimulate neighboring cells and enhance immune responses [9].

Extensive research has underscored the critical role of PANoptosis in tumor defense mechanisms. Studies have demonstrated that activation of the cGAS-STING pathway can induce PANoptosis within the tumor microenvironment. During this process, STING-mediated interferon signaling upregulates ZBP1 expression, facilitating the interaction between RIPK3 and CASP8, which culminates in PANoptosis induction. The susceptibility of STING-deficient mice to certain cancers, including breast and pancreatic tumors, highlights the pivotal role of the cGAS-STING pathway in regulating PANoptosis [10]. Recent studies have established clinical prognostic models for gastric, colorectal, and clear-cell renal carcinomas utilizing PANoptosis, further reinforcing its prognostic predictive potential in oncology [11–13].

In recent years, with the rapid development of high-throughput sequencing technology and bioinformatics, machine learning has played an increasingly important role in tumor immunology research [14]. Researchers have successfully constructed predictive molecular signature models by integrating multiomics datasets and employing various machine learning algorithms (such as random forest, support vector machine, and deep learning) to analyze sequencing data from purified immune cells, tumor cell lines, and tumor tissues [15]. As demonstrated in the studies by Zhang et al. and Liu et al. [16, 17], these computational methods not only assess patients' prognostic risk but also predict immunotherapy response and chemotherapy sensitivity. For instance, Zhang et al. utilized 10 machine learning algorithms (101 combinations) to identify immune features with predictive value in low-grade glioma, while Liu et al. confirmed in colorectal cancer research that machine learning–based prediction models showed superior predictive accuracy compared to traditional clinical variables in evaluating patients' overall survival, immunotherapy response, and personalized treatment selection. Through mining and analyzing massive biomedical data, machine learning algorithms can identify complex biomarker combinations, providing new research paradigms and technical support for precision medicine in cancer immunotherapy.

Nevertheless, the precise role of PANoptosis in LUAD immunotherapy and prognostic prediction remains to be fully elucidated. In this study, we aimed to develop a PANoptosis-based prognostic model for LUAD using multiomics data and systematically evaluate its clinical efficacy while investigating its correlation with clinical features, immune cell infiltration, and the tumor microenvironment. Through the integration of comprehensive bioinformatics analyses and machine learning algorithms, we constructed PANoptosis-related optimal model (PROM), a robust, accurate, and clinically applicable prognostic prediction tool for LUAD. This research not only provides novel insights into the mechanistic role of PANoptosis in LUAD progression but also offers practical methodologies for advancing precision medicine in LUAD treatment.

## 2. Method

### 2.1. Data Acquisition and Preprocessing

RNA sequencing profiles and clinical data were obtained from The Cancer Genome Atlas (TCGA) database, supplemented by six independent validation cohorts (GSE13213, GSE26939, GSE29016, GSE30219, GSE31210, and GSE42127) from the Gene Expression Omnibus (GEO). Raw expression counts were normalized to transcripts per million (TPM) using the following formula: TPM = (raw_count × 106)/(gene_length × sum(raw_count/gene_length)). Batch effects were mitigated using the ComBat algorithm (sva R package v3.42.0) [18, 19], with TCGA data serving as the reference batch and age, gender, and tumor stage as covariates. The normalized data underwent log2 transformation (log2[TPM + 1]) to enhance normality. Data quality was assessed through principal component analysis (PCA) using prcomp function with center = TRUE and scale = TRUE parameters, followed by sample-wise correlation analysis (Pearson correlation, threshold *r* > 0.8) to identify and remove potential technical outliers [20].

### 2.2. Single-Cell Transcriptomics Analysis

Single-cell RNA sequencing data from the GSE127465 dataset (> 30,000 cells) was processed using the Seurat v4.1.0 package [21, 22]. Quality control filters were applied to remove low-quality cells (unique gene count: 500–6000; mitochondrial gene percentage: < 20%; unique molecular identifier (UMI) count: 1000–30,000). Data normalization employed the LogNormalize method (scale.factor = 10,000), followed by identification of highly variable genes (*n* = 2000) using the FindVariableFeatures function (selection.method = “vst”). PANoptosis activity was quantified using AddModuleScore (nbin = 24, ctrl = 100, seed = 123), with scores scaled to a 0–1 range. Cell type–specific markers were identified using FindAllMarkers (min.pct = 0.25, logfc.threshold = 0.25, test.use = “wilcox”).

### 2.3. Intercellular Communication Network Analysis

Intercellular communication networks were constructed using CellChat v1.1.3 [23, 24], utilizing the default CellChatDB.human database containing 2021 validated ligand–receptor pairs. Expression data was normalized to counts per million (CPM) with a minimum expression threshold of 10% cells per group. Network inference was performed using probability calculations based on the law of mass action, with significance determined through 1000 permutation tests (*p* < 0.05). Communication strength was quantified as the product of interaction probability and average expression levels. Network centrality measures included degree centrality, betweenness centrality, and information flow centrality, calculated using the netAnalysis_computeCentrality function with default parameters.

### 2.4. PROM Development

PROM was developed through a systematic machine learning approach, beginning with univariate Cox regression (*p* < 0.05) for initial gene selection, requiring a minimum expression of TPM > 1 in 50% of samples. Ten machine learning algorithms were evaluated through tenfold cross-validation with 100 iterations: stepwise Cox (direction = “both,” criterion = “AIC”), Lasso (alpha = 1), ridge (alpha = 0, lambda = 10^seq(−10,10,100)), plsRcox (ncomp = 10), CoxBoost (stepno = 100), random survival forest (ntree = 1000, nodesize = 3), gradient boosting machine (ntrees = 1000, interaction.depth = 3), elastic net (alpha = 0.5), SuperPC (threshold = 0.4), and survival-SVM (kernel = “linear”). Model performance was assessed using Harrell's *C*-index, time-dependent area under the curve (AUC), and calibration plots, with stability evaluated through 1000 bootstrap iterations.

### 2.5. Functional and Pathway Enrichment Analysis

Gene Ontology (GO) and Kyoto Encyclopedia of Genes and Genomes (KEGG) analyses were performed using clusterProfiler v4.0.5 to elucidate the biological functions and pathways associated with relevant genes. Gene IDs were converted to Entrez IDs, and the Benjamini–Hochberg method was applied for multiple testing corrections (adjusted *p* < 0.05 considered significant). Gene set variation analysis (GSVA) was employed to investigate potential molecular mechanisms, with a focus on cancer-immune cycle and immunotherapy-related pathways. All pathway gene sets were sourced from the Molecular Signatures Database (MSigDB v7.4).

### 2.6. Immune Microenvironment Profiling

Immune microenvironment analysis integrated multiple computational approaches. Immunophenotype scores (IPS) were calculated using The Cancer Immunome Atlas (TCIA) [25] methodology, incorporating MHC molecules, immunomodulators, effector cells, and suppressor cells. Tumor immune dysfunction and exclusion (TIDE) algorithm was applied with LUAD-specific parameters to predict immune checkpoint blockade response. Immune cell infiltration was quantified using TIMER2.0, which integrates multiple algorithms (TIMER, xCell, EPIC, quanTIseq, and CIBERSORT) with cell type–specific gene signatures. A consensus score was calculated as the weighted average of normalized scores from each algorithm, with weights determined by algorithm performance in validation datasets.

### 2.7. Chemotherapy Response Prediction

Drug sensitivity prediction was performed using the pRRophetic package with the Genomics of Drug Sensitivity in Cancer 2 (GDSC2) database as reference. Gene expression data was preprocessed using ComBat adjustment and log2 transformation. Half-maximal inhibitory concentration (IC50) values were predicted using ridge regression (alpha = 0) with tenfold cross-validation. The analysis focused on standard-of-care chemotherapeutics for LUAD, with prediction accuracy assessed through root mean squared error (RMSE) and Pearson correlation between predicted and observed IC50 values in the Genomics of Drug Sensitivity in Cancer (GDSC) validation set.

### 2.8. Statistical Analyses

Statistical analyses were conducted using R v4.2.0. Survival analysis employed the Kaplan–Meier method with log-rank test, and Cox proportional hazards assumptions were verified using the cox.zph function. Group comparisons for continuous variables used either Student's *t*-test (for normal distributions, verified by Shapiro–Wilk test, *p* > 0.05) or Wilcoxon rank-sum test (for nonnormal distributions). Categorical variables were compared using chi-square test (expected frequency > 5) or Fisher's exact test (expected frequency ≤ 5). Correlation analyses used Spearman rank correlation with false discovery rate (FDR) correction for multiple testing. All statistical tests were two-sided, with significance threshold *p* < 0.05, and effect sizes were reported with 95% confidence intervals.

## 3. Result

### 3.1. Analysis of PANoptotic Activity and Immune Dynamics in LUAD Cells

**Figure 1:**
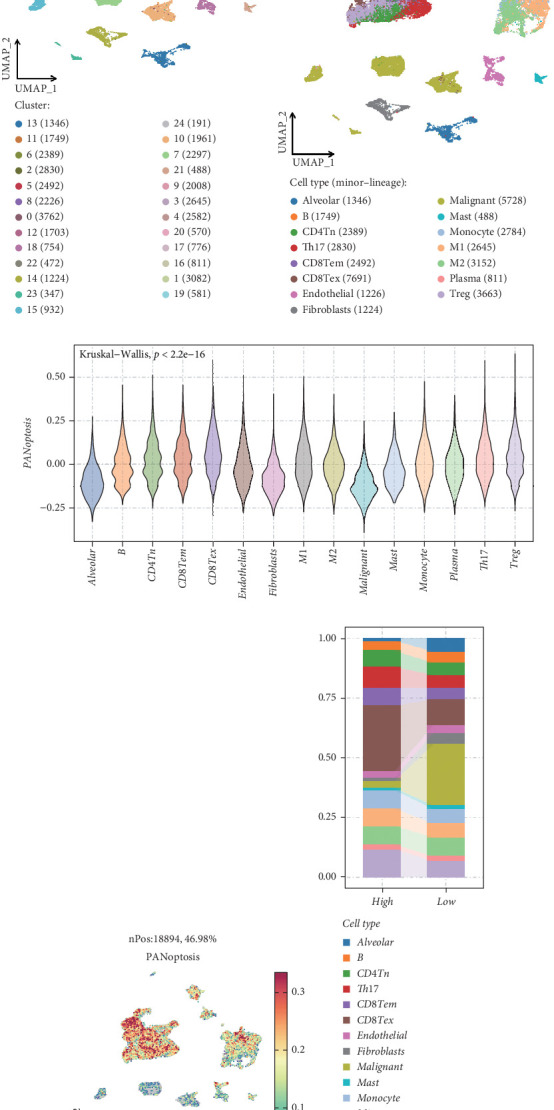
scRNA-seq data analysis. (a) Bubble plot demonstrating the differential expression patterns of canonical marker genes across distinct cellular populations, with dot size representing the percentage of cells expressing the gene and color intensity indicating the average expression level. (b) Unsupervised clustering analysis using uniform manifold approximation and projection (UMAP) revealed 25 distinct cell clusters, each represented by a unique color. (c) Comprehensive annotation of 15 distinct cell types based on the expression profiles of well-established lineage-specific marker genes, visualized through UMAP projection. (d) Violin plot illustrating the distribution and density of PANoptosis-related gene expression signatures across various cell populations, with width indicating the frequency of cells at each expression level. (e) UMAP visualization depicting the spatial distribution of PANoptosis scores across all identified cell populations, with color gradient representing score intensity. (f) Stacked bar plot showing the compositional changes in cellular populations across different PANoptosis score groups, represented as relative percentages of 15 annotated cell types. (g) Circular visualization comparing the frequency of intercellular communications between distinct cell clusters under high versus low PANoptosis score conditions, with connecting lines indicating interaction events. (h) Circular plot depicting the differential strength of cell–cell interactions between clusters under high versus low PANoptosis score conditions, with line thickness proportional to interaction strength.

In this study, we analyzed data from the Tumor Immune Single-cell Hub (TISCH) database focusing on LUAD, examining a total of 40,218 cells. Through clustering analysis, these cells were categorized into 19 distinct clusters. By analyzing the expression of classical marker genes, we successfully annotated 15 cell types (Figures 1(a), 1(b), and 1(c)). Additionally, we retrieved the PANoptosis gene set from the GeneCards database and applied the AddModuleScore algorithm to score each single cell, thus characterizing PANoptotic activity across different cell types (Figures 1(d) and 1(e)). Notably, CD8 T cells exhibited the highest PANoptotic scores, suggesting that this cell subset could play a critical role in mediating PANoptotic functions. Based on PANoptotic scores, we stratified the single cells into high- and low-expression groups. The bar chart indicated a significant reduction of CD8 T cell exhaustion (CD8 Tex) cells and an increase in malignant cells in the low-expression group (Figure 1(f)). This phenomenon may be due to the decreased number of CD8 T cells responsible for executing apoptotic functions, impairing their ability to efficiently target and destroy tumor cells, leading to tumor cell proliferation. Further analysis revealed significantly enhanced communication frequency and intensity among cell populations with high PANoptotic scores (Figures 1(g) and 1(h)). This could reflect a complex interaction network that enhances apoptotic capacity, highlighting the potential importance of CD8 T cells in tumor suppression through apoptotic mechanisms. This section elucidates the critical role of immune cell dynamics in cancer progression and identifies potential therapeutic targets for modulating immune responses in LUAD.

### 3.2. Enhanced Information Flow in Signaling Pathways of High PANoptosis Expression Group and Its Role in Immune Modulation

**Figure 2:**
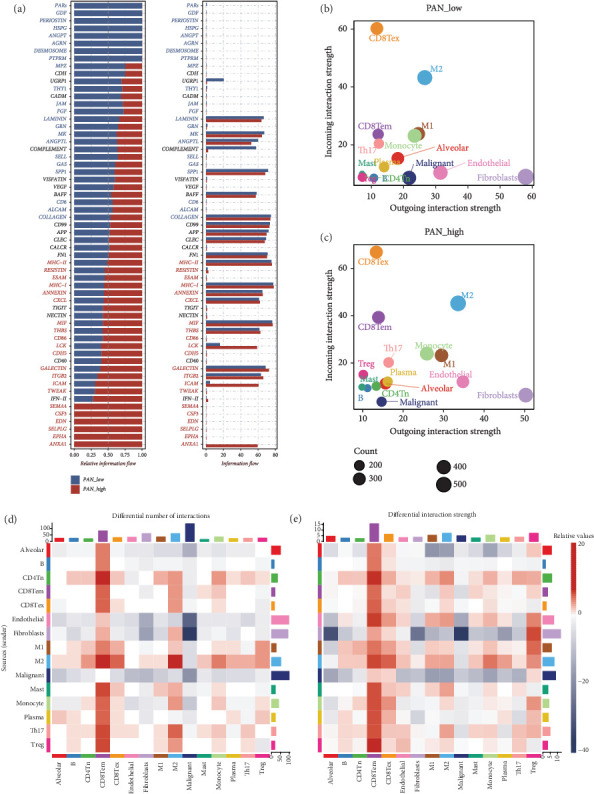
Cell communication analysis. (a) Comprehensive CellChat analysis revealing differential signaling pathway activation patterns between high- and low-PANoptosis groups. (b) Quantitative assessment of bidirectional signaling interactions in low-PANoptosis samples, illustrating both incoming (receiver) and outgoing (sender) signal strengths across distinct cellular populations. (c) Systematic analysis of cellular communication networks in high-PANoptosis samples, demonstrating the relative strengths of incoming and outgoing signals among different cell types. (d) CellChat analysis of differences in cell–cell interaction numbers between high- and low-PANoptosis samples. (e) CellChat analysis of differences in cell–cell interaction strengths between high- and low-PANoptosis samples.

We further investigated the differences in specific signaling pathways between different PANoptosis expression groups.  Figure 2(a) illustrates the relative (left) and absolute (right) information flow of intercellular signaling pathways in the high PANoptosis expression group (PAN_high) and the low PANoptosis expression group (PAN_low). The analysis reveals that pathways such as MHC-II, MHC-I, CXCL, ICAM, and CD86 exhibit significantly higher information flow in the PAN_high group, suggesting their critical roles in the PANoptosis process. Moreover, subsequent analyses indicate a marked increase in both outgoing and incoming signaling across nearly all cell types in the PAN_high group (Figures 2(b) and 2(c)). This phenomenon is further substantiated by additional evidence shown in Figure 2, which corroborates the pronounced enhancement of intercellular communication in response to heightened PANoptotic activity. These findings underscore the potential significance of these pathways in modulating immune responses and highlight their relevance as therapeutic targets in diseases characterized by dysregulated apoptotic mechanisms.

### 3.3. Identification and Characterization of PANoptosis-Related Genes With Prognostic Significance in LUAD

**Figure 3:**
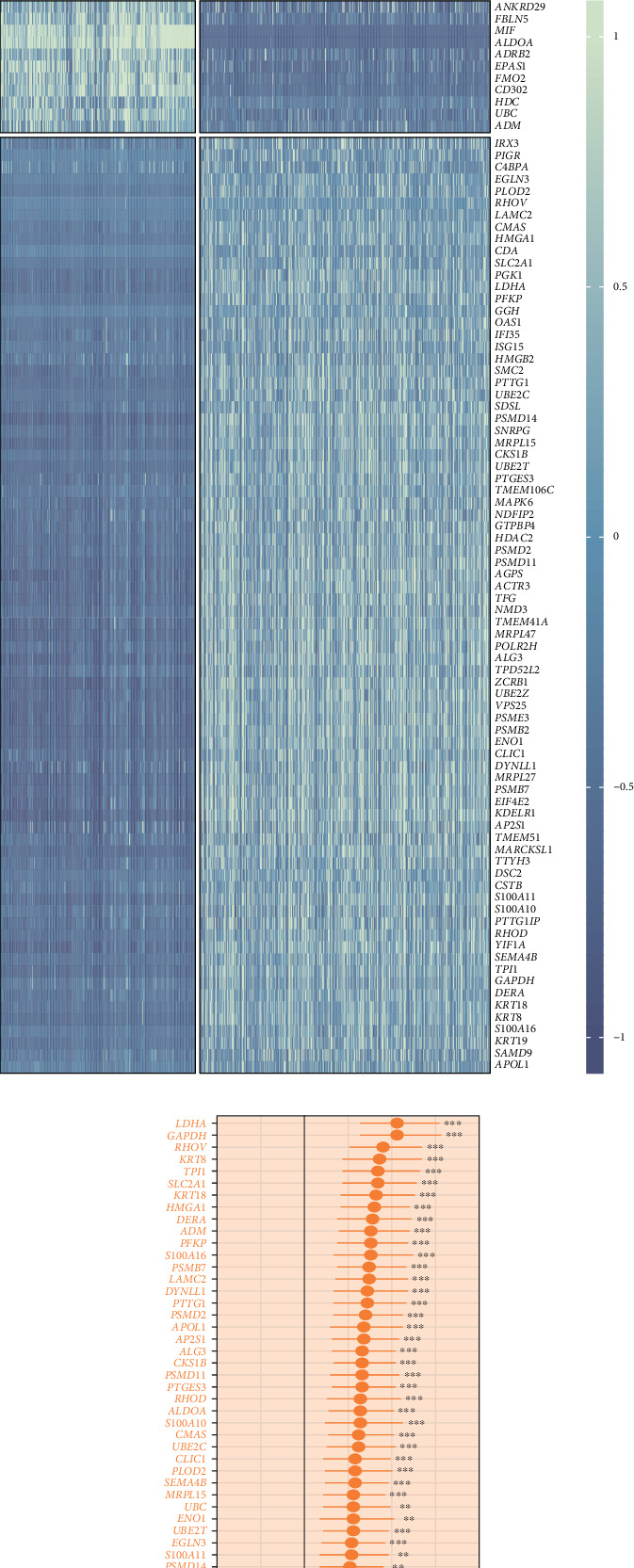
Identification of candidate genes for model construction. (a) Identification of 150 genes most correlated with PANoptosis scores for subsequent analysis. (b) Post–batch correction distribution of samples from seven LUAD datasets visualized via PCA. (c) Heatmap showing the expression of candidate genes in tumor versus normal samples. (d) Association of candidate genes with prognosis, where hazard ratios greater than 1 indicate risk factors, and those less than 1 indicate protective factors.

We employed Spearman correlation analysis to identify the top 150 genes most correlated with PANoptosis scores (Figure 3(a)). Subsequently, we utilized the findmarker function to explore differentially expressed genes between high- and low-PANoptosis groups. The integration of these two gene sets established a comprehensive set of PANoptosis-related genes. Next, we incorporated large-scale bulk RNA-seq data from patients with LUAD and performed batch correction to ensure data consistency (Figure 3(b)). Using Cox proportional hazards analysis along with differential expression analysis, we identified genes that not only displayed differential expression between tumor and normal tissues but also held prognostic significance. Ultimately, this led to the selection of a total of 89 genes with potential implications in cancer prognosis (Figures 3(c) and 3(d)).

### 3.4. Multiomics Characterization of PANoptosis-Related Genes in LUAD

**Figure 4:**
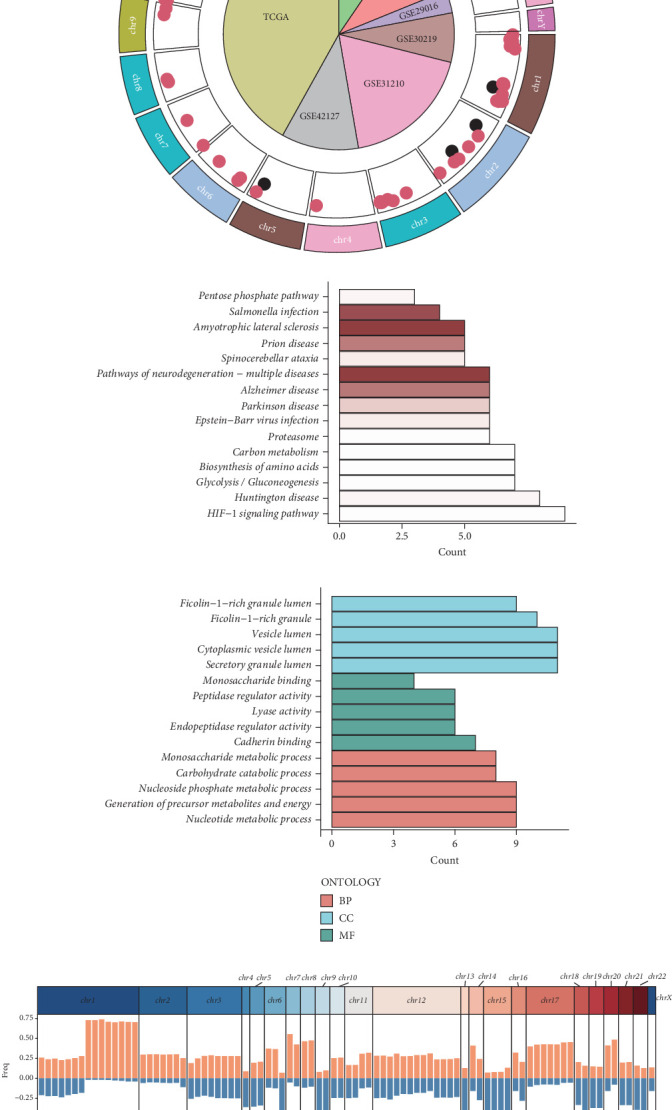
Functional enrichment and copy number variation distribution of candidate genes. (a) Composition of the LUAD datasets. (b) KEGG enrichment analysis of candidate genes, with a bar chart showing the top 15 significantly enriched pathways in LUAD. (c) Gene Ontology enrichment analysis of candidate genes, with a bar chart showing the top 15 significantly enriched pathways in LUAD. (d) Frequency distribution of copy number variations in candidate genes.

The chromosomal localization of the 87 differentially expressed genes is illustrated in Figure 4(a). The outermost circle delineates chromosome numbers, while the central scatter plot depicts the 89 key PANoptosis-related genes. Red dots signify upregulation in tumor tissue, whereas black dots indicate downregulation. The inset pie chart represents the distribution of samples across seven LUAD datasets utilized in subsequent analyses, with slice sizes proportional to sample quantities. Figures 4(b) and 4(c) elucidate the KEGG and GO pathway enrichment profiles of these genes, respectively. Notably, the predominant enriched pathways encompass metabolic processes, vesicular and secretory granule functions, enzymatic activities, and binding functionalities. These pathways are pivotal in cellular metabolism, intracellular transport, and signal transduction, all of which are critical for neoplastic transformation and progression. Specifically, the enriched metabolic pathways, such as the pentose phosphate pathway and carbon metabolism, underscore the altered metabolic state characteristic of cancer cells, often referred to as the Warburg effect. The enrichment in vesicular and secretory granule functions suggests potential roles in exosome-mediated intercellular communication and tumor microenvironment modulation. Enzymatic activities and binding functions point towards alterations in cellular signaling cascades and protein–protein interactions, which may contribute to aberrant cell proliferation and survival.  Figure 4(d) illustrates the copy number variation (CNV) alterations of these genes, highlighting genomic instability as a hallmark of cancer. The CNV profile provides insights into potential gene dosage effects and chromosomal aberrations that may drive oncogenesis. Understanding these genetic alterations is crucial for identifying potential driver mutations and developing targeted therapies.

### 3.5. Application of Machine Learning–Based PROM in LUAD Prognosis Prediction

**Figure 5:**
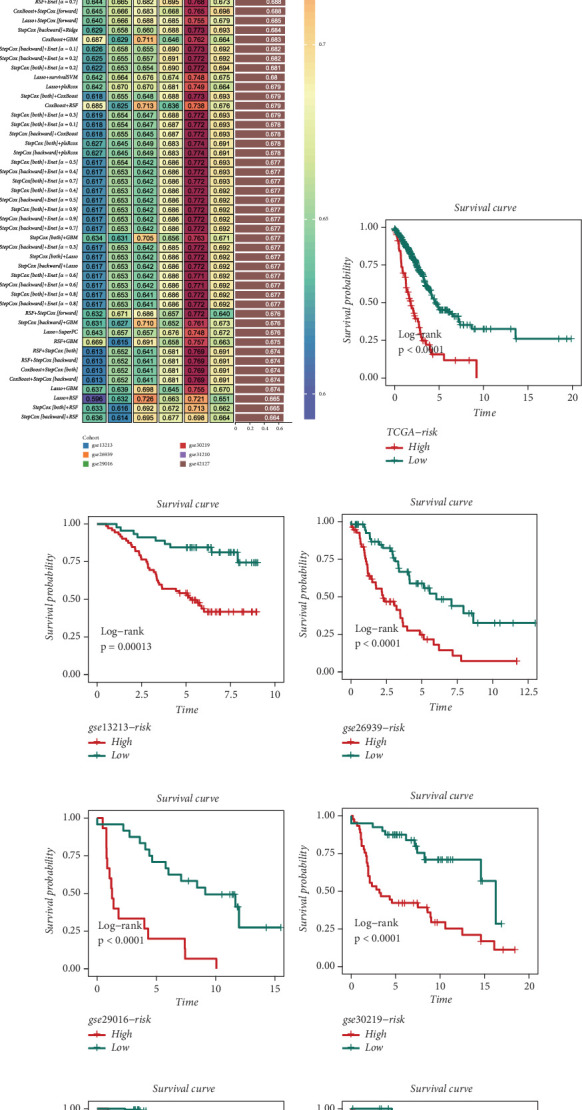
Development of a risk model using machine learning across multiple LUAD datasets. (a) Heatmap illustrating the performance of risk models developed by various machine learning algorithm combinations in LUAD datasets, evaluated using the mean *C*-index. (b–h) Kaplan–Meier curves assessing the predictive performance of the best model across different LUAD datasets, where high-risk samples exhibit poorer survival rates.

Utilizing an ensemble of 10 machine learning algorithms, we performed a comprehensive model comparison to identify the optimal predictive framework. As illustrated in Figure 5(a), the Lasso + Stepcox combination emerged as the superior model, demonstrating the highest concordance index (*C*-index) of 0.698. This hybrid approach leverages the feature selection capabilities of Lasso regression with the time-to-event modeling of stepwise Cox regression, resulting in a robust prognostic tool. To validate the prognostic efficacy of our model, we conducted extensive cross-validation across seven independent cohorts. The results consistently demonstrated the discriminative power of our PROM in stratifying patient outcomes. As depicted in Figures 5(b), 5(c), 5(d), 5(e), 5(f), 5(g), and 5(h), the PROM consistently differentiated patients into high- and low-risk groups across all validation cohorts, with the high-PROM group invariably exhibiting significantly poorer prognosis (log-rank test, *p* < 0.05 for all cohorts).

### 3.6. Robust Predictive Performance and Biological Relevance of the PROM

**Figure 6:**
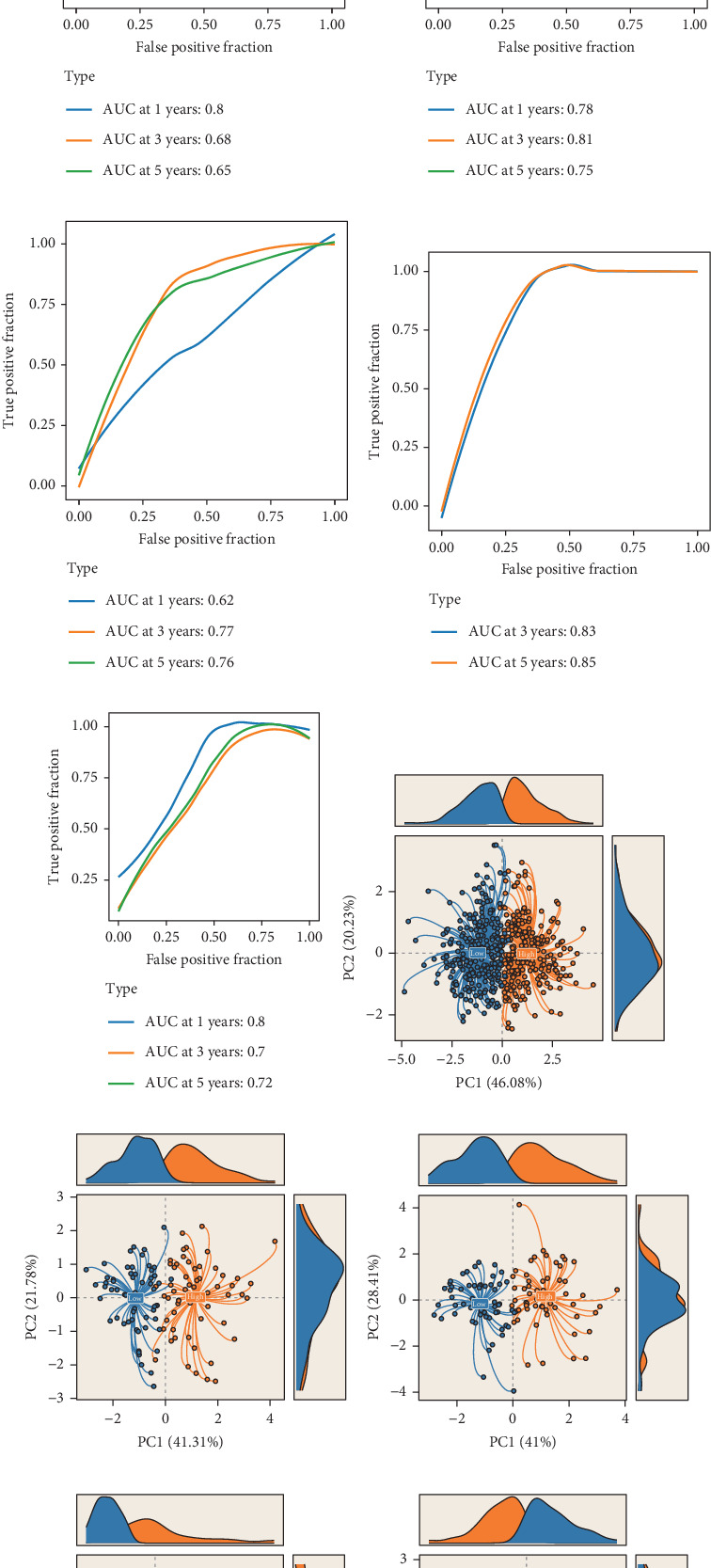
Evaluation of the predictive ability of the risk model. (a–g) Time-dependent ROC curves evaluating the predictive performance of the model, with AUC values as the evaluation metric. (h–n) PCA analysis assessing the model's ability to distinguish between sample groups.

To further evaluate the discriminatory power of PROM, we performed receiver operating characteristic (ROC) curve analyses. The results demonstrated exceptional predictive accuracy, with AUC values exceeding 0.7 in all seven cohorts (Figures 6(a), 6(b), 6(c), 6(d), 6(e), 6(f), and 6(g)). Notably, in several cohorts, the AUC surpassed 0.8, indicating excellent discriminative ability. These findings highlight the model's consistency and reliability across diverse patient populations. To gain deeper insights into the molecular basis of PROM's prognostic capabilities, we conducted PCA using the expression profiles of the model's constituent genes. Strikingly, PCA revealed a consistent ability to stratify patients into two distinct clusters across all seven LUAD datasets (Figures 6(h), 6(i), 6(j), 6(k), 6(l), 6(m), and 6(n)). This clear separation based on gene expression patterns provides strong evidence for the biological relevance of the PROM signature and its ability to capture fundamental differences in tumor biology that correlate with patient outcomes.

### 3.7. PROM Score Predicts Immunotherapy Response in LUAD: Integrative Analysis of Tumor Immune Microenvironment

The TCIA revealed significantly higher TCIA scores in the low-PROM group (Figure S1A), suggesting a more favorable immune microenvironment for immunotherapy in this cohort. This finding indicates that LUAD patients with low PROM scores may be more likely to benefit from immune checkpoint inhibitors and other immunotherapeutic approaches. To further elucidate the relationship between PROM and the tumor microenvironment, we employed the Estimation of STromal and Immune cells in MAlignant Tumor tissues using Expression data (ESTIMATE) algorithm. Our analysis uncovered a striking inverse correlation between PROM scores and both immune and stromal scores, while demonstrating a positive correlation with tumor purity (Figure S1B). These results suggest that LUAD tumors with high PROM scores are characterized by a less immunologically active microenvironment and potentially reduced infiltration of immune and stromal cells. Building upon these findings, we conducted a TIDE analysis to assess potential responses to immune checkpoint inhibition. Intriguingly, the high-PROM group exhibited significantly elevated TIDE scores (Figure S1C), indicative of a higher likelihood of immune dysfunction or exclusion. This observation aligns with the TCIA results and further supports the notion that LUAD patients with high PROM scores may be less responsive to immunotherapy. Moreover, our analysis revealed a substantially larger proportion of potential immunotherapy responders within the low-PROM group (Figure S1D).

### 3.8. PROM Score as a Predictor of Immune Infiltration and Drug Sensitivity in LUAD: Insights From TCGA and Drug Screening Analyses

Utilizing the TIMER 2.0 database, we extracted immune infiltration evaluation data from TCGA tumor datasets. Our analysis revealed that the low-PROM group exhibited significantly higher immune cell infiltration, including T cells, B cells, and other immune cell subsets (Figure S2A). This finding suggests that PROM scores may serve as a potential indicator of the tumor immune microenvironment, with lower scores associated with a more immunologically active milieu. To identify potentially effective therapeutic agents targeting different PROM groups, we conducted a comprehensive drug screening analysis. Our results unveiled several promising candidates with lower IC50 values in the high-PROM group, including BMS-536924 (an IGF-1R inhibitor), alisertib (an Aurora kinase A inhibitor), cytarabine (a nucleoside analog), 5-fluorouracil (a pyrimidine analog), foretinib (a multikinase inhibitor), and gemcitabine (a nucleoside metabolic inhibitor) (Figure S2B). These findings suggest that tumors with high PROM scores may be more sensitive to these agents, potentially offering tailored treatment strategies for this subgroup of patients. Subsequently, we employed single-sample gene set enrichment analysis (ssGSEA) to explore differential pathway activities between PROM groups. Our results demonstrated a strong positive correlation between PROM scores and the activation of multiple signaling pathways, including cell cycle progression, DNA replication, and other proliferation-associated processes. Conversely, PROM scores showed a notable negative correlation with pathways involved in B cell and CD4+ T cell recruitment (Figure S2C).

## 4. Discussion

PANoptosis, a recently characterized form of regulated cell death, has demonstrated significant pathophysiological implications across diverse disease states. Current investigations into PANoptosis have predominantly focused on infectious diseases, where its pivotal role in host defense mechanisms has been well established. This molecular pathway efficiently eliminates infected cells and restricts pathogen dissemination through the activation of the PANoptosome complex, which orchestrates the integration of pyroptosis, apoptosis, and necroptosis signaling cascades [26]. However, dysregulated or excessive activation of PANoptosis may precipitate disease onset and progression, with pathogens potentially exploiting this pathway to enhance their survival, subsequently leading to excessive host inflammatory responses, severe tissue injury, or systemic inflammatory response syndrome (SIRS). Moreover, PANoptosis exhibits crucial involvement in autoimmune disorders, which are characteristically manifested by aberrant immune responses and chronic inflammation. Through the amplification of proinflammatory signaling pathways, PANoptosis may exacerbate the pathogenesis and progression of these conditions [27]. For instance, in systemic lupus erythematosus (SLE) and rheumatoid arthritis (RA), aberrant inflammasome activation has been demonstrated to trigger PANoptosis, thereby potentiating tissue damage and perpetuating inflammatory cascades.

Recent studies have revealed tumor-related biomarkers associated with PANoptosis. Caspase-1, a crucial enzyme in this process, plays a key role in the assembly of inflammasomes and the subsequent activation of cytokines like IL-1*β* and IL-18 [28]. In a range of cancers, including colorectal and breast cancer, the activation of Caspase-1 has been linked to inflammatory responses and a less favorable prognosis [29–31]. Increased expression of Caspase-1 serves as a marker for heightened tumor aggression and poor prognoses. The NLRP3 inflammasome, a key player in inflammatory responses, tends to be overactive in specific types of cancer, such as liver and lung cancers. This overactivation is intricately linked to the advancement of tumors. Moreover, elevated levels of NLRP3 may correlate with the proliferation of tumor cells and their ability to withstand therapeutic interventions [32]. GSDMD plays a pivotal role in the process of PANoptosis, inducing cell death by compromising cell membranes via its N-terminal domain. In various types of cancer, such as breast and pancreatic cancers, the activation of GSDMD has been associated with both the tumor's immune microenvironment and the effectiveness of treatments. Additionally, the level of GSDMD expression may influence the invasiveness of tumor cells [29, 33, 34]. cGAS serves as an intracellular sensor for DNA, playing a pivotal role in promoting PANoptosis by stimulating the STING pathway [35]. Within cancer cells, the engagement of the cGAS/STING pathway is often linked to immune evasion and resistance to therapeutic agents, where varying levels of cGAS expression can affect how tumors respond to immune checkpoint inhibitors [36]. Although the investigation of PANoptosis in cancer research is still in its preliminary stages, bioinformatics analysis is anticipated to offer valuable insights and guidance for future fundamental and clinical studies [37].

This comprehensive study elucidates the complex interplay between PANoptotic activity, immune dynamics, and clinical outcomes in LUAD. The elevated PANoptotic scores observed in CD8+ T cells indicate their crucial role in mediating PANoptotic functions within the tumor microenvironment, while the negative correlation between PANoptotic activity and malignant cell prevalence highlights potential tumor-suppressive effects. Enhanced intercellular communication in high PANoptotic score populations suggests a complex network of cell death mechanisms. The upregulation of MHC-II, MHC-I, CXCL, ICAM, and CD86 pathways in high PANoptotic expression groups indicates potential mechanisms for immune modulation and therapeutic targeting. The identification of 89 PANoptosis-related genes with prognostic significance, enriched in metabolic processes, vesicular functions, and enzymatic activities, provides a molecular signature enhancing risk stratification.

Based on these findings, we conducted a comprehensive model comparison through an ensemble analysis of 10 machine learning algorithms to determine the optimal predictive framework. The Lasso + Stepcox combination emerged as the superior model, achieving a *C*-index of 0.698. This hybrid approach combines the feature selection capabilities of Lasso regression with the time-event modeling advantages of stepwise Cox regression, forming a robust prognostic tool. In extensive cross-validation across seven independent cohorts, our PROM demonstrated significant prognostic discriminative ability, effectively stratifying patients into high- and low-risk groups, with the high PROM group consistently showing significantly poorer prognosis (log-rank test, *p* < 0.05 for all cohorts).

PROM's consistent performance across multiple analytical methods (ROC analysis and PCA) provides compelling evidence for its reliability as a LUAD prognostic biomarker. The clear stratification observed in PCA indicates that PROM captures key biological features differentiating between high- and low-risk tumors. These observations reveal the biological basis of PROM's prognostic predictive capability, suggesting that high PROM scores may be associated with enhanced proliferative capacity and reduced immune activity in the tumor microenvironment. The negative correlation between PROM scores and immune cell recruitment pathways aligns with TIMER 2.0 analysis results, reinforcing PROM's crucial role in modulating the tumor immune landscape.

In clinical applications, the negative correlation between PROM scores and immune infiltration, along with differences in TIDE and TCIA scores, suggests PROM's potential as a biomarker for predicting immunotherapy response and guiding treatment decisions. The strong correlation between PROM scores and cell cycle–related pathways indicates that targeting these processes may be particularly effective in high-PROM tumors, consistent with the lower IC50 values observed for cell cycle–targeting agents in the high-PROM group. The differential drug sensitivity between PROM groups provides a theoretical basis for personalized treatment approaches, particularly the enhanced sensitivity of high-PROM tumors to drugs like BMS-536924 and alisertib, suggesting new avenues for targeted intervention.

In conclusion, the development of PROM as a robust prognostic tool, coupled with its potential to predict immunotherapy response and guide treatment selection, represents a significant advancement in personalized medicine for LUAD. This model not only provides accurate prognostic prediction but also offers new perspectives and evidence for clinical treatment decision-making.

## Data Availability

The complete set of data utilized in this investigation can be obtained from two primary repositories: The Cancer Genome Atlas (TCGA), which is accessible via http://cancergenome.nih.gov/, and the Gene Expression Omnibus (GEO), available at https://www.ncbi.nlm.nih.gov/geo/. These publicly available databases house all the relevant datasets employed throughout our study.

## Supporting Information

Supporting Information 1 Figure S1: Potential of the risk model in predicting immunotherapy outcomes. (A) IPS scores used to predict differences in immunotherapy response between high- and low-risk samples. (B) Scatter plot showing the correlation between model scores and stromal score, immune score, ESTIMAT score, and tumor purity. (C, D) TIDE analysis evaluating differences in immunotherapy response between high- and low-risk samples.

Supporting Information 2 Figure S2: Immune infiltration characteristics and drug sensitivity analysis in high- and low-risk groups. (A) Heatmap illustrating differences in immune cell infiltration between high- and low-risk samples, estimated using seven different algorithms. (B) Boxplot displaying differences in sensitivity to eight drugs between high- and low-risk samples. (C) ssGSEA evaluating the correlation between risk scores and pathways involved in immunotherapy and the tumor immune cycle.
