# Supplementary figures and images for: PANoptosis-Related Optimal Model (PROM): A Novel Prognostic Tool Unveiling Immune Dynamics in Lung Adenocarcinoma
Source: Int J Genomics. 2025 Feb 18;2025:5595391. doi: 10.1155/ijog/5595391 (PMC11858721; doi:10.1155/ijog/5595391)

A

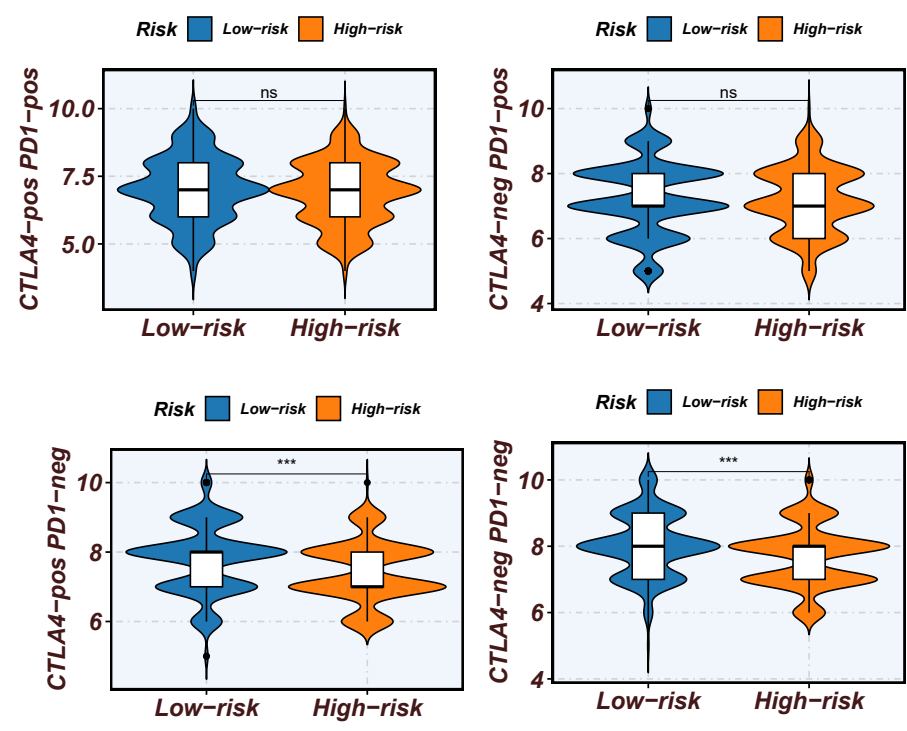

C

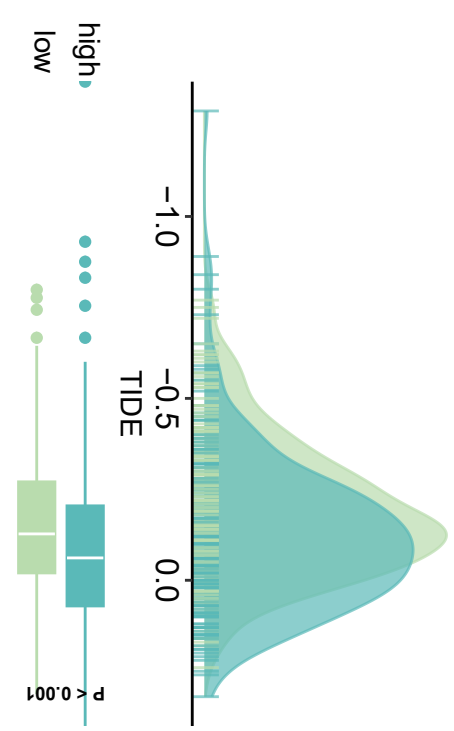

B

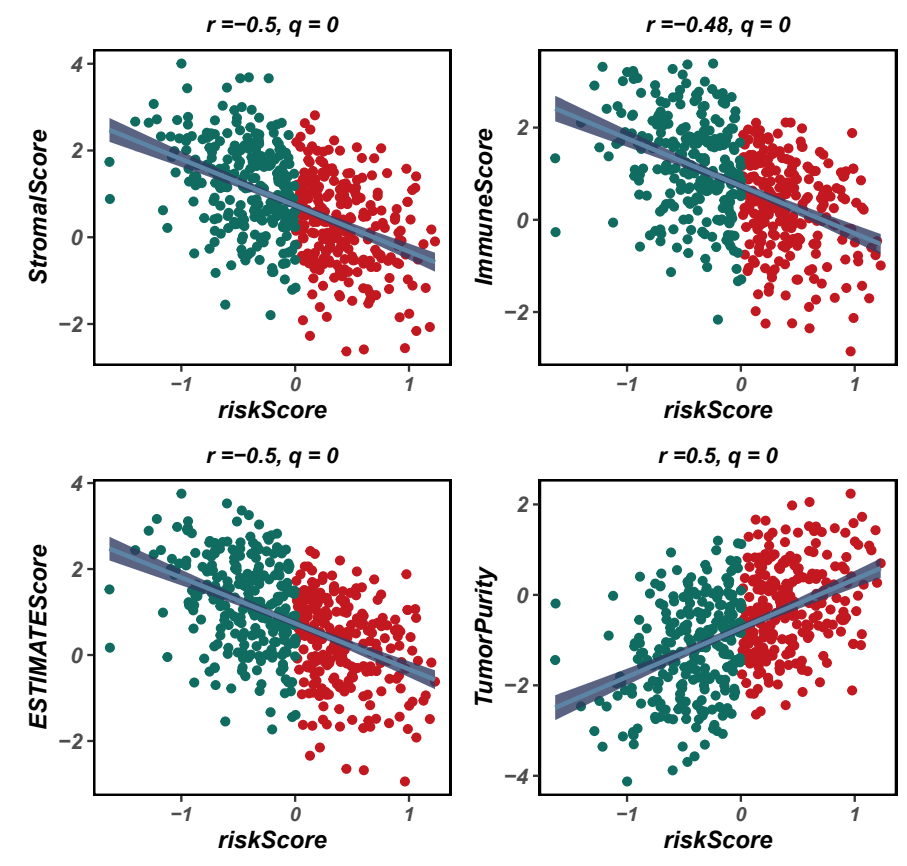

D

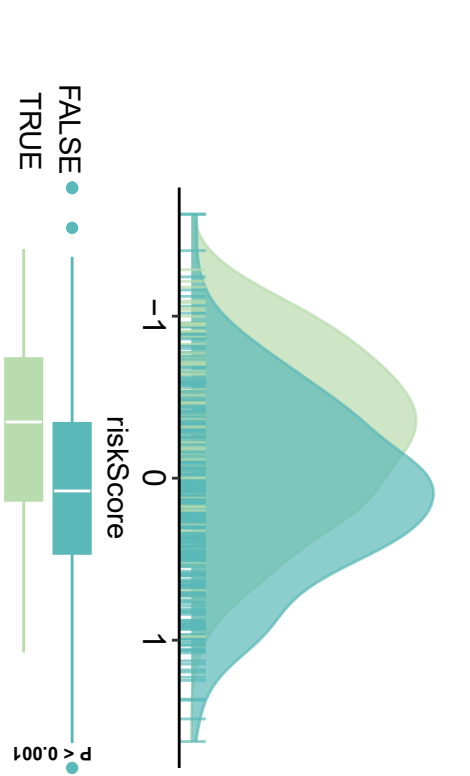

Supplement: Supporting Information 1 — Figure S1: Potential of the risk model in predicting immunotherapy outcomes. (A) IPS scores used to predict differences in immunotherapy response between high- and low-risk samples. (B) Scatter plot showing the correlation between model scores and stromal score, immune score, ESTIMAT score, and tumor purity. (C, D) TIDE analysis evaluating differences in immunotherapy response between high- and low-risk samples. [file 5595391.f1.pdf]

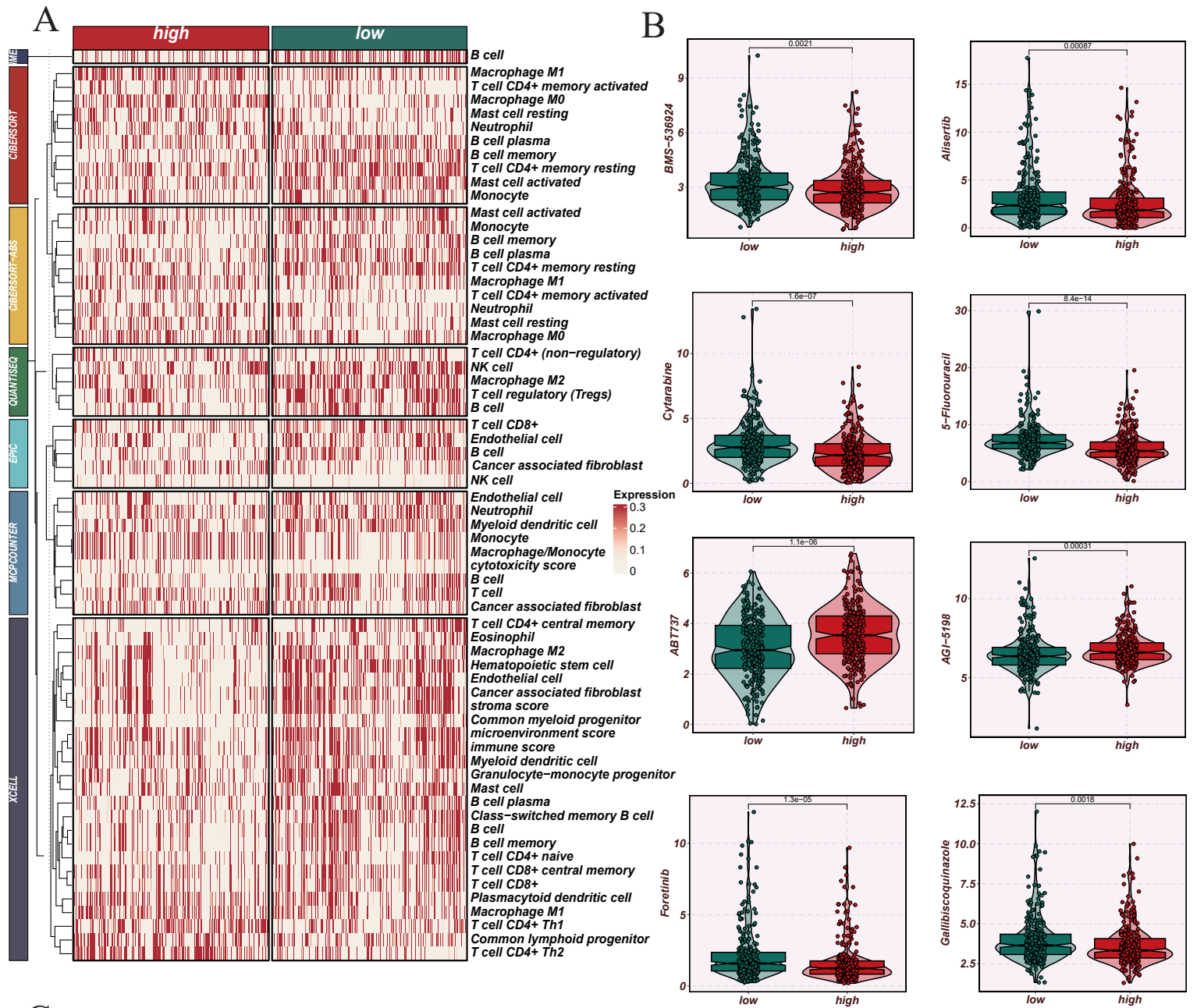

Supplement: Supporting Information 2 — Figure S2: Immune infiltration characteristics and drug sensitivity analysis in high- and low-risk groups. (A) Heatmap illustrating differences in immune cell infiltration between high- and low-risk samples, estimated using seven different algorithms. (B) Boxplot displaying differences in sensitivity to eight drugs between high- and low-risk samples. (C) ssGSEA evaluating the correlation between risk scores and pathways involved in immunotherapy and the tumor immune cycle. [file 5595391.f2.pdf]
